# Oral hygiene influence on the incidence and severity of oral manifestations in Coronavirus Disease 2019

**DOI:** 10.1186/s12903-025-06075-2

**Published:** 2025-05-21

**Authors:** Başak Yılmaz Çınar, Oya Türkoğlu, Sema Becerik

**Affiliations:** 1 Private practice, Bursa, Turkey; 2 Department of Periodontology, Ege University, School of Dentistry, Bornova, İzmi̇r, 35100 Turkey

**Keywords:** Covid 19, Taste dysfunction, Xerostomia, Aphthous lesions, Halitosis

## Abstract

**Intro:**

The aim of this study was to evaluate the incidence, severity, duration of oral manifestations in individuals with Coronavirus Disease 2019 (COVID-19) and the association of these manifestations with the severity of COVID-19 and the patient's oral hygiene.

**Methods:**

This study included 820 patients with confirmed COVID-19. A questionnaire form including oral hygiene habits, the severity of Covid-19, the presence, severity and durations of oral manifestations was prepared, and a web-based survey was performed using Google-forms. Obtained data was analysed with Pearson chi-square and Fisher's exact tests with statistical significance set at *P* < 0.05.

**Results:**

The most commonly reported manifestations were taste dysfunction (63.4%), xerostomia (59.9%), halitosis (31.1%), dysphagia (27.8%), hypersensitive teeth (27.2%) and gingival bleeding (14.3%). The incidence of the oral manifestations was found significantly associated with severity of COVID-19 (*P* = 0.000 V = 0.151), presence of systemic diseases (*P* = 0.034, V = 0.074) and age (*P* = 0.023, V = 0.099). Tooth brushing decreased the incidence of aphthous like lesions of tongue during Covid-19 (*p* < 0.05).

**Conclusion:**

Maintenance of oral hygiene was associated with a reduced incidence of aphthous-like lesions, underscoring the protective role of routine oral care. These findings highlight the need to integrate oral health assessment and hygiene education into COVID-19 management protocols, which may also be important for potential future pandemics.

## Introduction

Acute respiratory syndrome coronavirus 2 (SARS-CoV-2) is a novel human coronavirus responsible for the Coronavirus Disease 2019 (COVID-19) which was declared as a pandemic by the World Health Organization (WHO) on March 2020 [1]. According to the data of the WHO, as of 23 June 2024, over 775 million confirmed cases and more than seven million deaths have been reported globally since the beginning of pandemic [2].

COVID-19 manifests a broad spectrum of symptoms ranging from asymptomatic infection, to mild and self-limited symptoms, to severe disease requiring hospitalization, and to excessive immune activation and cytokine storm leading to multiple organ failure, shock, and death [3, 4]. Common symptoms of the disease are fever, cough, fatigue, slight dyspnea, sore throat, headache and conjunctivitis [5, 6]. Other COVID-19 complications may include acute liver, cardiac and kidney injury, as well as secondary infection and inflammatory response [5, 6].

SARS-COV-2 virus primarily targets the respiratory system but several studies have suggested that the virus can also affect oral and periodontal health [7–9]. Wide array of oral signs and symptoms were reported in COVID-19 patients [10]. Smell and taste loss symptoms were added to COVID-19 screening protocols in the very early pandemic [11]. Taste alterations serve as one of the key COVID-19 symptoms [12] and the prevalence of taste alterations was reported in nearly 50% to %65 of all COVID-19 patients [10, 13].

In the recent reviews and meta-analyses, the oral findings of COVID-19 and the plausible causal relationship of oral lesions with COVID-19 were evaluated [14–16]. It has been proposed that oral lesions could be complications of the disease or related to medical care or pre-existing medical conditions of the patients. Studies have detected the presence of the virus in the saliva and oral mucosa of COVID-19 patients, indicating that the virus can replicate in the oral cavity [17, 18]. It was suggested that the SARS-CoV-2 virus could directly infect the oral tissues and lead to oral lesions such as ulcers or blisters [19]. Moreover, due to the severity of COVID-19 symptoms the patient could face difficulty in achieving oral hygiene which may led to oral lesions. The lack of oral hygiene in severe cases of COVID-19 could cause oral lesions especially in intubated patients. Trauma secondary to intubation, coexisting medical conditions such as diabetes or immunosuppression, vascular complications, and opportunistic or secondary infections should be considered as other potential confounding factors for the oral lesions COVID-19 patients [20, 21].

Periodontal diseases are the infectious disease caused by microorganisms in dental plaque. Dental plaque removal by regular oral hygiene habits is the key factor for preventing oral diseases. Multiple studies have associated COVID-19 severity with viral load [22, 23]. High viral loads are present not only in the nasal cavity and nasopharynx but also in the oropharynx, making the oral cavity a significant reservoir for infectious viruses. In the present study, we hypothesized that regular tooth brushing and interdental cleaning habits might prevent the appearance of oral manifestations and/or alleviate them. It was also hypothesized that the severity of COVID-19 or smoking might increase the severity of oral lesions. Although COVID-19 is no longer a global threat, examining its oral manifestations in relation to oral hygiene habits and disease severity can provide valuable insights for developing healthcare strategies to prepare for potential future outbreaks.

To date, many case reports [7], case series [19] and reviews [14–16] have been published reporting the oral manifestations of COVID-19, but there are limited number of studies on the factors affecting the prevalence and severity of oral manifestations in patients with COVID-19. The aims of this survey study were 1) to determine what the oral manifestations are and their incidence, severity, and duration in individuals with COVID-19, 2) to investigate the association of these symptoms with the severity of COVID-19 and the patient's oral hygiene habits, 3) to determine the association between the presence of systemic diseases and the occurrence of oral manifestations 4) to evaluate whether smoking is a confounding factor for oral lesions in patients with COVID-19.

## Materials and methods

The sample size calculation indicated that a minimum of 384 participants would be required to achieve a 95% confidence level with a ± 5% margin of error. The questionnaire used in this study was adapted from previously published studies [24, 25]. A web-based survey was performed using Google forms from January to May 2021. A link on Google Drive was shared with the volunteer participants through the social media platforms used by the researchers. This study included 820 patients with the polymerase chain reaction (PCR) confirmed SARS-CoV-2 infection. Since this was a survey study, only the participants who were willing to answer the questionnaire were included in the study. Those who were not willing to answer the survey were excluded from the study. A text explaining the aim of the study was send to each participant before entering the study and informed consent in accordance with Helsinki declaration was obtained from each participant. The study protocol was approved by the Ethics Committee of the Ege University (ethics approval number:21–3.1 T/40).

The questionnaire was subdivided into four sections and consisted of mostly yes/no questions and some multiple-choice questions. In the first part of the questionnaire, questions addressed age, gender, smoking habit and presence of systemic disease of the participants.

The second part of the questionnaire included questions regarding the severity of COVID-19 and oral hygiene habits. The severity of COVID-19 were classified as; a) Asymptomatic to mild (presence of very limited symptoms) b) Moderate (bed rest at home because of symptoms like fever, cough, fatigue ext.) c) Severe (Hospitalized because of severe symptoms) d) Critical (Hospitalized and intubated). The classification of the disease severity was determined according to WHO [26]. The frequency of toothbrushing and interdental cleaning before COVID-19 and during COVID-19 were asked.

In the third part, questions concerning oral manifestations, periodontal and oral health during COVID-19 were asked. The questions addressed the presence of gingival bleeding, redness and swelling, presence of oral aphthous-like lesions and location of the lesions. The presence of herpes lesions, dental hypersensitivity, halitosis, dysgeusia, xerostomia (the subjective feeling of oral dryness), dysphagia and timing of these symptoms were also asked.

In the last part of questionnaire, the participants were asked if they could have accessed enough information about oral problems during the COVID-19 and if they have visited dentist after recovery of COVID-19.

### Statistical analysis

Responses to the survey were coded in an electronic spreadsheet and statistical analyses were performed by using IBM SPSS Statistics 25.0 (IBM SPSS Statistics for Windows, Version 25.0. Armonk, NY: IBM Corp.) The level of significance was determined as 0.05 in all of the analyses. Categorical variables were summarized as frequency and percentage. Quantitative variables were summarized as mean (M) and standard deviation (SD). Pearson Chi-square test or Fisher's exact probability test were used to evaluate the relationship between variables. Chi-square results were calculated as the effect size of Cohen's w with the formula $w=\sqrt{{\chi }^{2}/N}$. It is classified as w = 0.10 small, w = 0.30 medium, and w = 0.50 large effect size [27].

## Results

### Characteristics of the participants

A total of 820 COVID-19 patients including 577 (70.4%) females and 243 (29.6%) males were involved in the study. Among the participants, 321 (39.1%) of them were between the ages of 18 and 25, 350 (42.7%) were between the ages of 26 and 45, 135 (16.5%) were between the ages of 46 and 65 and 14 (1.7%) were over the age of 65. Regarding the smoking habits, 646 (78.8%) participants were non-smokers, 109 (13.3%) smoke less than 10 cigarettes per day, 53 (6.5%) smoke 10–20 cigarettes per day and 12 (1.5%) smoke more than 20 cigarettes per day. Among the participants 126 (15.4%) had systemic disease including hypertension (48, 5.9%), diabetes (27, 3.3%), cardiovascular disease (24, 2.9%) and other systemic diseases (27, 3.3%) (Table 1).

**Table 1:**
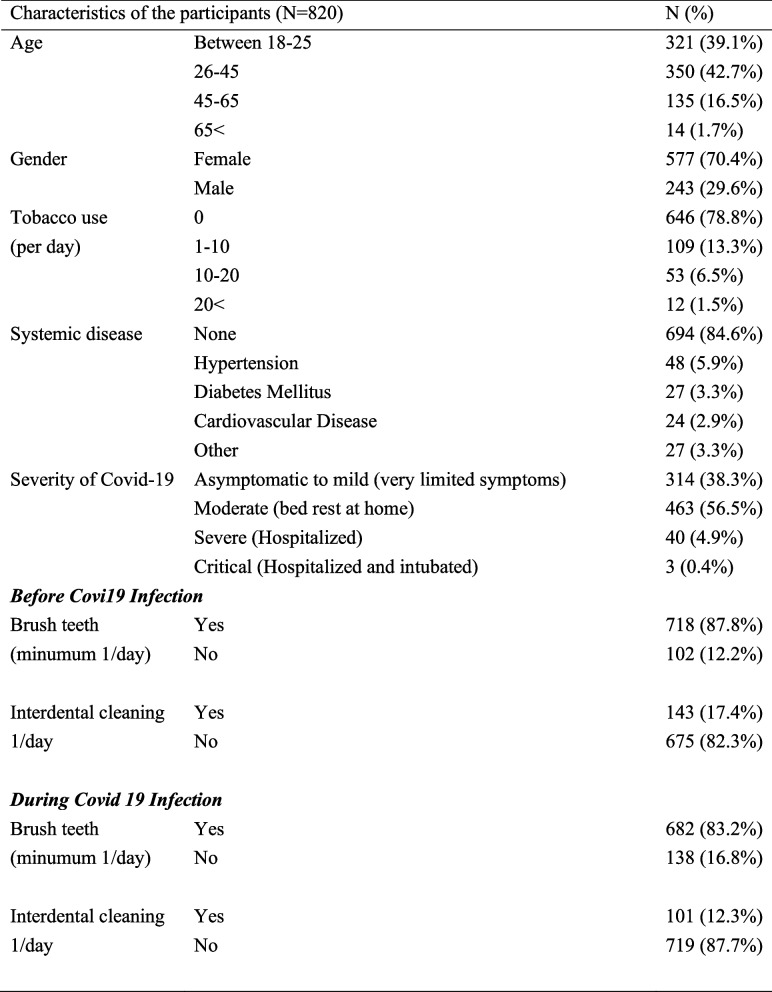
Characteristics of the participants

Regarding the severity of COVID-19; 314 (38.3%) participants were asymptomatic to mild, 463 (56%) were moderate, 40 (4.9%) were severe and 3 (0.4%) were critical. Amongst the participants 718 (87.6%) reported brushing their teeth at least once a day before COVID-19, while during COVID-19 the number of participants who could brush their teeth at least once a day decreased to 682 (%83.2) (*P* > 0.05). The number of patients who performed interdental cleaning (floss, interdental brush) once a day was 143 (17.4%) before COVID-19 and was 101 (12.3) during COVID-19 (*P* > 0.05) (Table 1).

### Oral manifestations during COVID-19

The frequency of oral manifestations in study participants was presented in Table 2. Regarding the gingival problems, the participants mostly expressed gingival bleeding (*n* = 117, 14.3%) followed by gingival redness (115, 14%) and gingival swelling (75, 9.1%). Apthous like lesions were reported to seen in 167 (20.4%) participants of the study. Locations of the apthous like lesions were mostly the buccal mucosa (*n* = 84, %36.1) followed by tongue (*n* = 62, 26.6%), gingiva (*n* = 54, 23.1%) and palate (*n* = 14, 2%). Pain caused by apthous like lesion were very severe in 8 (4.2%), severe in 23 (12.2%), mild in 62 (32.6%), and light in 54 (28.4%) participants. Forty-three (22.6%) participants had apthous like lesions with no pain. The appearance of the oral lesions was before the diagnoses of COVID-19 in 145 (32.3%) participants.

**Table 2:**
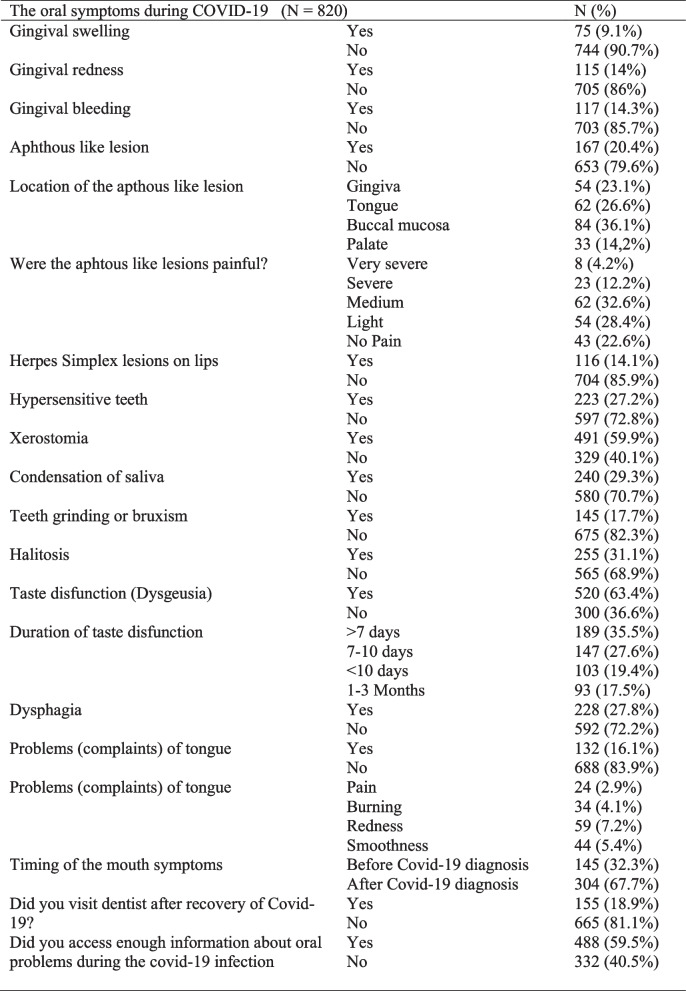
The oral symptoms during COVID-19 infection

The most reported symptoms were taste dysfunction (*n* = 520, 63.4%;), xerostomia (*n* = 491, 59.9%), and halitosis (*n* = 255, 31.1%). Condensation of saliva (*n* = 240, 29.3%), dysphagia (*n* = 228, 27.8%), hypersensitive teeth (*n* = 223, 27.2%;), and teeth grinding (bruxism) (*n* = 145, 17.7%) were also reported by the participants. Amongst participants, 132 (16.1%) of them experienced tongue problems including pain (*n* = 24, 2.9%), burning (*n* = 34, 4.1%), redness (*n* = 59, 7.2%) and smoothness (*n* = 44, 5.4%). One hundred and fifty-five (18.9%) participants visited dentist after the recovery of COVID-19 and 332 (40.5%) participants reported that they could not access enough information about oral problems during the COVID-19.

The effects of tooth brushing and interdental cleaning on aphthous lesions in patients with COVID-19 were presented in Table 3. Tooth brushing decreased the incidence of aphthous lesions of the tongue during COVID-19 (*P* < 0.05) while interdental cleaning decreased aphthous lesions in palate (*P* < 0.05). Table 4 shows the effect of smoking on oral lesions. No significant association was found between smoking and occurrence of oral lesions during COVID-19 in the present study (*P* > 0.05). The occurrence of aphthous lesions in gingiva were reported in the 3.7% of the participants under age 25, 8.6% of the participants between age 26–45, 7.4% of the participants between age 45–65 and 14.3% of the participants above 65 years old. The occurrence of aphthous lesions in gingiva increased significantly with age (*P* = 0.023 V = 0.099).

**Table 3:**
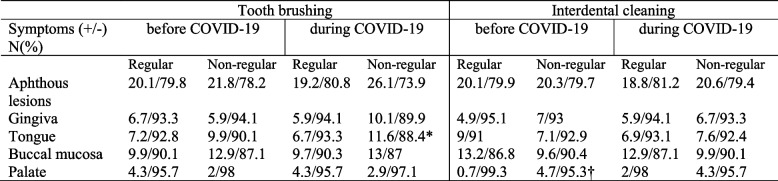
The effect of oral hygiene habits on presence of aphthous lesions

*P=0.049 V=0.069 significant difference in aphthous lesions in tongue between regular and non-regular tooth brushing during COVID-19 infection

†P=0.025 V=0.078 significant difference in aphthous lesions in palate between regular and non-regular interdental cleaning during COVID-19 infection

**Table 4:**
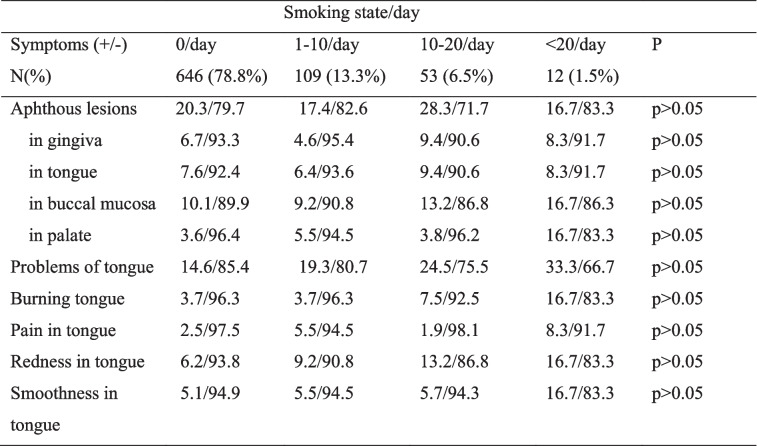
The effect of smoking state on oral lesions in patients with COVID-19 infection

The severity of COVID-19 was found associated with the incidence of the oral lesions (*P* = 0.000 V = 0.151) (Table 5). As the severity of COVID-19 worsens from mild to severe, more lesions on tongue (*P* = 0.002 V = 0.130) and buccal mucosa (*P* = 0.000 V = 0.155) were reported by the participants. The severity of COVID-19 found associated with the complaints of the tongue (*P* = 0.001 V = 0.116) including pain (*P* = 0.044 V = 0.084), redness *P* = 0.005. V = 0.003 and smoothness (*P* = 0.022 V = 0.049). The severity of COVID-19 was found associated with sore in the throat (*P* = 0.007 V = 0.023), and herpes lesions of lip (*P* = 0.001 V = 0.004). Xerostomia (*P* = 0.000 V = 0.000) and loss of taste was also found associated with the severity of COVID-19 (*P* = 0.000 V = 0.000).

**Table 5:**
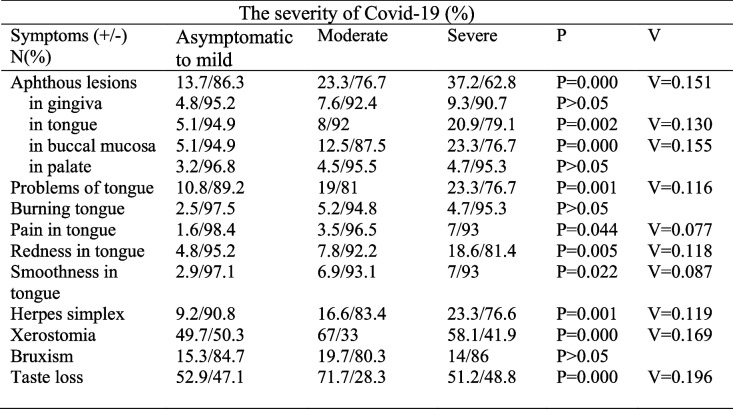
The effect of the severity of COVID-19 infection and oral lesions

The occurrence of oral lesions during COVID-19 was found to be associated with the presence of systemic diseases in the present study (*P* = 0.034, V = 0.074). The effects of diabetes, cardiovascular diseases and hypertension on incidence of oral lesions during COVID-19 were presented in Table 6. Aphthous lesions on the palate were significantly more reported by the participants with diabetes (*P* = 0.004, V = 0.101) than the participants without systemic disease. The participants with cardiovascular diseases reported to have more aphthous lesions in gingiva (*P* = 0.067 V = 0.071) and palate (*P* = 0.000 V = 0.185). No relationship was found with hypertension and occurrence of oral manifestations during COVID-19 (*P* > 0.05).

**Table 6:**
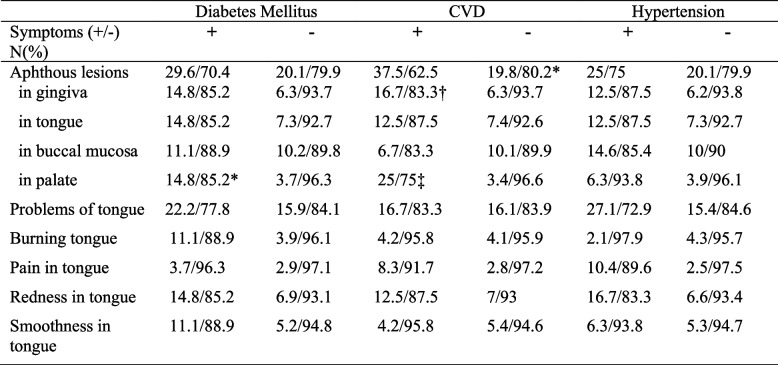
The effects of systemic diseases on oral lesions. The percentage of patients presenting oral lesions with and without systemic disease

*CVD* Cardio Vascular Diseases

*P=0.004 V=0.101 significantly higher aphthous lesions in palate in patients with Diabetes Mellitus than those of non-Diabetes Mellitus during COVID-19 infection

†P=0.067 V=0.071 significantly higher aphthous lesions in gingiva in patients with CVD than those of non-CVD during COVID-19 infection

‡P=0.000 V=0.185 significantly higher aphthous lesions in palate in patients with CVD than those of non-CVD during COVID-19 infection

## Discussion

In the present study, taste dysfunction was the most common finding reported during COVID-19, which was observed in 63.4% of the participants. The duration of taste dysfunction was extended 1–3 months in 11% of the participants. In accordance with the current study, taste dysfunction during COVID-19 was observed 52% of 140 participants by Biadsee [28] and 60% of 111 participants by Fantozzi et al. [29] The second most observed symptom was xerostomia and observed in 59.9% of the participants in the current study. In a survey study involving 665 COVID-19 patients it was reported that 47% of them manifested xerostomia [25]. Saliva is a fundamental component for the maintenance of oral health and decreased saliva production may led to problems, including changes in taste, dysphagia, halitosis, dental caries, impaired use of prostheses and recurrent infections [30]. Accordingly, halitosis was manifested by 31.1% of the COVID-19 patients in the current study and dysphagia was reported by 27.8% of them. The pathophysiologic mechanism and association of these manifestations in COVID-19 are still controversial. SARS-CoV-2 might infect salivary gland, impair salivary quality and flow leading to xerostomia, taste disorders, and halitosis [28, 31]. Hyposalivation is the main etiologic factor of xerostomia but it has been suggested that the association with medications, nasal congestion and mouth breathing, nutritional deficiency, diabetes, and the anxiety might also play a role in xerostomia [28, 25, 32].

In the literature, there are not many studies evaluating gingival complaints during COVID-19. In the current study 14.3% of the participants reported gingival bleeding, 14% of them had gingival redness and 9.1% had gingival swelling. In a pilot study conducted by El-Kady et al. [33] the prevalence of gingival bleeding was found 7% in 58 COVID-19 patients. In a case series study published at the beginning of the pandemic, the oral findings of three COVID-19 patients were presented and severe gingival bleeding was suggested to be associated with COVID-19 [34]. The virus may alter the immune response in the mouth, leading to the overproduction of inflammatory cytokines that can damage oral tissue [35] and also systemic inflammation caused by COVID-19 can exacerbate existing oral health problems [36]. Additionally, during COVID-19, patients may not maintain proper oral hygiene due to the stress and systemic weakness caused by the disease. The increased accumulation of dental biofilm causing an enhanced inflammatory response and clinical signs of gingivitis and/or periodontitis might be one explanation of the enhanced gingival complaints.

In the present study 14.1% participants had herpes simplex lesions on their lips. Among the 820 participants of the current study, 20.4% of them manifested aphthous lesions in oral cavity. In previous studies oral ulceration was reported to be manifested in 20.4% of 573 COVID-19 patients by Ebu bakır [25] and 17.2% of 58 COVID-19 patients by El-Kady et al. [33] Pain caused by oral lesions varies from very severe to light and 22,6% of the participants had oral lesions with no pain in this current study. If oral lesions are painless, patients may not be aware of these lesions. Therefore we consider that the prevalence of aphthous lesions might be higher than indicated in survey studies. Locations of the aphthous oral lesions were mostly the buccal mucosa, tongue, gingiva, palate, and throat, respectively in the present study. Similar to the results of our study the most commonly involved oral sites have been reported to be tongue, palate, lips, gingiva, and buccal mucosa [10, 16]. It was suggested that SARS-CoV-2 could infect oral epithelial cells directly through ACE2 receptors, which are highly expressed by oral epithelial cells [37, 38]. Other factors like anxiety, stress, medications might contribute to the development and progression of oral lesions such as recurrent aphthous ulcers in COVID-19 patients [16, 25].

The appearance of the oral lesions was before the diagnoses of COVID-19 in 17.7% participants of the current study. It was suggested that oral infection provides an early detection and transmission window that may precede symptom onset in COVID-19 [39]. In a prospective study diagnosis of oral lesions were confirmed by oral examination and laboratory confirmation in 123 COVID-19 patients and suggested that early oral lesions showing peripheral thrombosis on histopathological analysis could be a warning sign of possible progression to severe disease [40]. Oral health care professionals should be aware that these oral manifestations can play role in early diagnosis and monitoring of the COVID-19.

In the present study tooth brushing decreased the incidence of aphthous lesions of the tongue during COVID-19. In line with the current study, significantly increased oral ulcerations and dental/oral pain were reported in the patients with decreased oral hygiene measures during COVID-19 [25]. This can be attributed to plaque and calculus accumulation around the teeth in case of poor oral hygiene, which will subsequently lead to the inflammation and ulceration of the gingiva, and eventually cause periodontitis, orofacial pain and tooth loss. This finding is confirmed by a study which investigated the complications of COVID-19 in patients with poor oral health. The authors reported that there was a link between elevated bacterial loads in the oral cavity and post-viral complications, and improving oral hygiene measures could decrease the risk of COVID-19 complications [25]. Kamel et al. [24] reported that participants with poor oral health had significantly higher CRP levels than those with good oral health. It was suggested that good oral hygiene could help reduce viral load and secondary bacterial infections, thereby decreasing COVID-19 severity.

No significant association was found between smoking and occurrence of oral lesions during COVID-19 in the present study. However, the high proportion of non-smokers in our study population (78.8%) and the low prevalence of heavy smokers (only 1%) may have contributed to the inability to detect the potential adverse effects of smoking. El Tantawi et al. [41] reported that gingival inflammation, xerostomia, change in taste, burns and ulcers and hairy tongue presence was not different between smokers and non-smokers in COVID-19 infected young adults. The occurrence of oral lesions during COVID-19 was found to increase with age in the current study. Similarly, Sabbagh et al. [42] stated that the relation between COVID-19 and change in taste and xerostomia was affected by age in adolescents and young adults.

The occurrence of oral lesions during COVID-19 was found to be associated with the presence of systemic diseases in the present study especially diabetes and cardiovascular diseases. This result is also consistent with other studies reporting oral lesions could be caused by the effects of systemic diseases [43]. Diabetes, and cardiovascular diseases have been recognized as risk factors for COVID-19 infection and are associated with increased disease severity [44]. While these systemic conditions were shown to contribute to COVID-19-related oral manifestations in this study, further research is needed to clarify their exact role as contributing factors. The severity of COVID-19 was found to be associated with the incidence of the oral lesions. As the severity of COVID-19 worsens from mild to critical more lesions on tongue and buccal mucosa were reported by the participants. The severity of COVID-19 was also associated with the complaints of the tongue, herpes lesions of lip, xerostomia and loss of taste in the present study.

COVID-19 is highly contagious and requires quarantine of infected persons, very few studies have been conducted and published that directly address the question of the prevalence of oral manifestations in patients with COVID-19 using adequate detection methods. Many case reports and case series have been published reporting oral manifestations of COVID-19 which conducted on a limited number of patients or have usually included hospitalised severe patients. There are studies using patient-reported questionnaires as a data collection tool [25, 28], as in this study. This survey study has some limitations. It was not possible to perform an oral examination of the patients, and the clinical features of the oral lesions could not be professionally observed. The absence of clinical oral examinations is a major limitation of the study. The subjective nature of self-reported symptoms and potential recall bias may further affect data accuracy. Additionally, the lack of standardized diagnostic criteria for COVID-19-related oral lesions limits comparability across studies. It was not possible to determine which virus variant the patients were infected with, and to assess whether there was a difference in oral manifestations between the different variants. This study consisted mainly of patients with mild to moderate COVID-19 and is not representative of the entire COVID-19 population, especially severe cases. Drugs used for COVID-19 treatment and their association with oral lesions observed during COVID-19 were not evaluated.

It can be concluded that the occurrence of oral manifestations during COVID-19 was found to increase with age, presence of systemic diseases, increase with the severity of COVID-19 and poor oral hygiene. Individuals who maintain good oral hygiene appear to experience fewer oral complications compared to those with poor oral care. These findings highlight the importance of integrating oral health assessments and hygiene education into COVID-19 management protocols, which could also contribute to minimizing oral complications in future pandemics. It could be suggested that infected patients should be informed about the oral manifestations of COVID-19 disease especially patients which also have systemic diseases.

### Clinical relevance

Oral manifestations of COVID-19 increases with severity of COVID-19, age, presence of systemic disease and poor oral hygiene. It can be said that people who can maintain proper oral hygiene might have fewer oral health problems if they get COVID-19 compared to people who do not maintain proper oral hygiene.

## Data Availability

The data sets used and/or analysed during the current study are available from the corresponding author on request.

## Abbreviations

SARS-CoV-2: Acute respiratory syndrome coronavirus 2
COVID-19: Coronavirus Disease
WHO: World Health Organization
PCR: Polymerase chain reaction
M: Mean
SD: Standard deviation
